# Attraction of Ethiopian phlebotomine sand flies (Diptera: Psychodidae) to light and sugar-yeast mixtures (CO_2_)

**DOI:** 10.1186/1756-3305-6-341

**Published:** 2013-12-05

**Authors:** Oscar D Kirstein, Roy Faiman, Araya Gebreselassie, Asrat Hailu, Teshome Gebre-Michael, Alon Warburg

**Affiliations:** 1 Department of Microbiology and Molecular Genetics, The Institute for Medical Research Israel-Canada, The Kuvin Centre for the Study of Infectious and Tropical Diseases, The Hebrew University – Hadassah Medical School, The Hebrew University of Jerusalem, Jerusalem 91120, Israel; 2 Aklilu Lemma Institute of Pathobiology, Addis Ababa University, Addis Ababa, Ethiopia; 3 Department of Microbiology, Immunology & Parasitology, Faculty of Medicine, Addis Ababa University, Addis Ababa, Ethiopia; 4 Present Address: Department of Entomology, Cornell University, Ithaca, NY 14853, USA

**Keywords:** Attractants for blood-sucking insects, Chemical light-stick, Ethiopia, *Phlebotomus orientalis*, *Sergentomyia*, Visceral leishmaniasis, Yeast fermentation

## Abstract

**Background:**

Visceral leishmaniasis (VL) known as Kala-Azar is a serious systemic disease caused by *Leishmania donovani* parasites (Trypanosomatidae: Kinetoplastida). The disease is prevalent in the Indian Sub-continent, East Africa and Brazil. In Africa, the worst affected regions are in Sudan, with an estimated 15,000-20,000 cases annually and Ethiopia with 5,000-7,000 cases a year. The main vector of VL in Sudan and Northern Ethiopia is *Phlebotomus orientalis,* a sand fly frequently found in association with *Acacia spp* and *Balanite spp* woodlands.

**Methods:**

To optimize sampling of sand flies for epidemiological studies in remote areas we tested different means of attraction. Miniature suction traps employing 2AA batteries (3 V) were deployed in the up-draft orientation and baited with chemical light-sticks (Red, Yellow and Green), or bakers’ yeast in sugar solution (emitting CO_2_ and perhaps other attractants). These traps were compared with standard CDC incandescent light traps employing 6 V batteries. Trials were conducted during two consecutive years at different localities around Sheraro, a town in West Tigray, Northern Ethiopia.

**Results:**

The sand fly species composition was similar but not identical in the different locations tested with different *Sergentomyia* spp. predominating. *Phlebotomus* spp. comprised less than 1% of the catches during the first year trials (November – December 2011) but increased markedly during the second year trials performed later in the dry season at the height of the sand fly season in February 2012. Although there did not appear to be a species bias towards different light wave-lengths, fermenting yeast in sugar solution attracted relatively more *Phlebotomus* spp. and *Sergentomyia schwetzi*.

**Conclusions:**

Although the standard 6 V CDC incandescent light traps captured more sand flies, light-weight (~350 g) 3 V suction traps baited with chemical light-sticks were shown to be effective means of monitoring sand flies. Such traps operated without light and baited with yeast in sugar solution caught relatively more *Phlebotomus* spp.

## Background

Phlebotomine sand flies (Diptera: Psychodidae) are of considerable public health importance in many regions of the world where they transmit the causative agents of leishmaniasis, bartonellosis and sand fly fever [1-5]. The leishmaniases are a group of diseases caused by parasites of the genus *Leishmania,* affecting mainly the poorest of the poor in developing countries; 350 million people are considered at risk of contracting leishmaniasis, and some two million new cases occur yearly [6,7]. The proven vectors of leishmaniasis in the old world belong to the genus *Phlebotomus* Rondani & Berté, 1840 and new world vectors to the genus *Lutzomyia* Franca & Parrot, 1920 [1,8,9].

Visceral leishmaniasis (VL) or Kala-Azar, caused by infection with *Leishmania donovani* complex parasites is prevalent in 65 countries. The majority (90%) of cases occur in poor rural and suburban areas of 5 countries: Bangladesh, India, Nepal, Sudan and Brazil [7,10]. In East Africa VL is widely distributed throughout much of Sudan, the lowland regions of Ethiopia as well as Kenya and South Sudan [7,11,12]. In Ethiopia, important endemic foci include the Humera and Metema plains in northwestern Tigray and the Segen and Woyto river valleys in the southwest [11,13].

The sand fly *Phlebotomus (Larroussius) orientalis* Parrot, 1936 is the main vector of VL in Sudan and in Northern Ethiopia where it is frequently associated with *Acacia seyal* and *Balanites aegyptiaca* woodlands growing in black cotton soils [14-16]. *P. orientalis* is primarily a lowland species, but in Ethiopia it is also found in altitudes of up to 2,000 m [17]. In the more southerly VL foci, *P. orientalis, P. martini* and *P. celiae* have been implicated as vectors of *L. donovani*[18-20].

Sand flies belonging to the genus *Sergentomyia* are the predominant phlebotomine species in many African ecosystems [21-23]. *Sergentomyia* spp. are known to transmit *Leishmania* that infect lizards (previously *Sauroleishmania*) [24]. Although most species are preferential reptile-feeders certain species also bite mammals including humans and were suspected of transmitting human leishmaniasis [25,26]. In our study area, *Sergentomyia* spp. were exceptionally abundant and some species were found to feed on humans (Moncaz *et al.* unpublished). This finding, coupled with the almost total absence of *Phlebotomus* spp. at certain times of the year, warranted the inclusion of *Sergentomyia* spp. in the current study despite their dubious role as vectors of leishmaniasis to humans [25,27].

Centers for disease control (CDC) miniature light traps that are frequently used for collecting sand flies attract sand flies from short distances not much exceeding two meters [28]. CDC traps baited with carbon dioxide (CO_2_) have been used extensively for trapping blood-sucking insects and are considered superior to CDC light traps for both mosquitoes and sand flies. Moreover, CO_2_-baited traps have the added advantage that most moths and beetles are not attracted by CO_2_ so the yield is usually considerably “cleaner” [29-32]. CO_2_ for baiting traps is routinely supplied from either pressurized cylinders fitted with pressure valves or from dry ice [29,30]. Such traps typically emit CO_2_ at concentrations that are roughly two orders of magnitude higher than background levels (20,000 vs. 400 ppm) [33]. Blood seeking female mosquitoes and sand flies are generally thought to respond to small changes in CO_2_ concentration by flying upwind and maneuvering between concentration gradients and visual cues until reaching their target [30,34]. Unfortunately, neither dry ice nor pressurized gas cylinders are easily found in remote areas of developing countries where many leishmaniasis foci are found. Sugar solutions fermented by Bakers’ yeast (sugar/yeast mixtures [SYM]) were demonstrated to be attractive to Anopheline mosquitoes in Tanzania [35]. We have previously demonstrated the efficacy of both SYM and chemiluminescent light-sticks as attractants for phlebotomine sand flies in Ethiopia and in Israel using horizontal sticky traps [36]. Here, we combined these attractants with inverted up-draft suction traps similar to CDC traps but fabricated in our laboratory and designed to operate on 2 AA type rechargeable batteries (3 V). Such traps weigh less than 350 g each making it possible to carry and deploy a large number in areas where vehicle access is restricted. These 3 V traps are not equipped with incandescent lights because 2 AA batteries do not provide sufficient power to run both motor and light for 12 hours. Therefore, to experiment with light traps we utilized chemiluminescent light-sticks to attract the sand flies to the 3 V traps [36].

The current study forms part of a comprehensive project on the ecology and transmission dynamics of visceral leishmaniasis in Ethiopia. The experiments reported here were conceived and designed to optimize and facilitate large scale trapping of sand flies using equipment and materials appropriate for remote areas. At the same time, the results comprise the only entomological data pertaining to this important focus of kala azar in the Sheraro district of northern Ethiopia.

## Methods

### Experimental sites

The study was carried out during the dry seasons of 2011–12 in rural environments near the town of Sheraro (14° 24′ 09.69″N – 37° 46′ 39.69″E). Sheraro town is a sub-district of the Tahtay Adiabo district, located in the Semien Mi’irabawi Zone (Northwestern) of Tigray Region, northern Ethiopia at an elevation of circa 1,000 m above sea level. The climate is semi-arid typical of the African Sahel, with an extended dry period of nine to ten months with most all of the rains (~600 mm) concentrated in 50 to 60 days during June-September. The mean annual temperature is ~ 38°-40°C year round [37]. The landform comprises shallow valleys of black-cotton soils normally used for planting sorghum interspersed with sandy and rocky outcrops upon which the villages are built.

**Figure 1:**
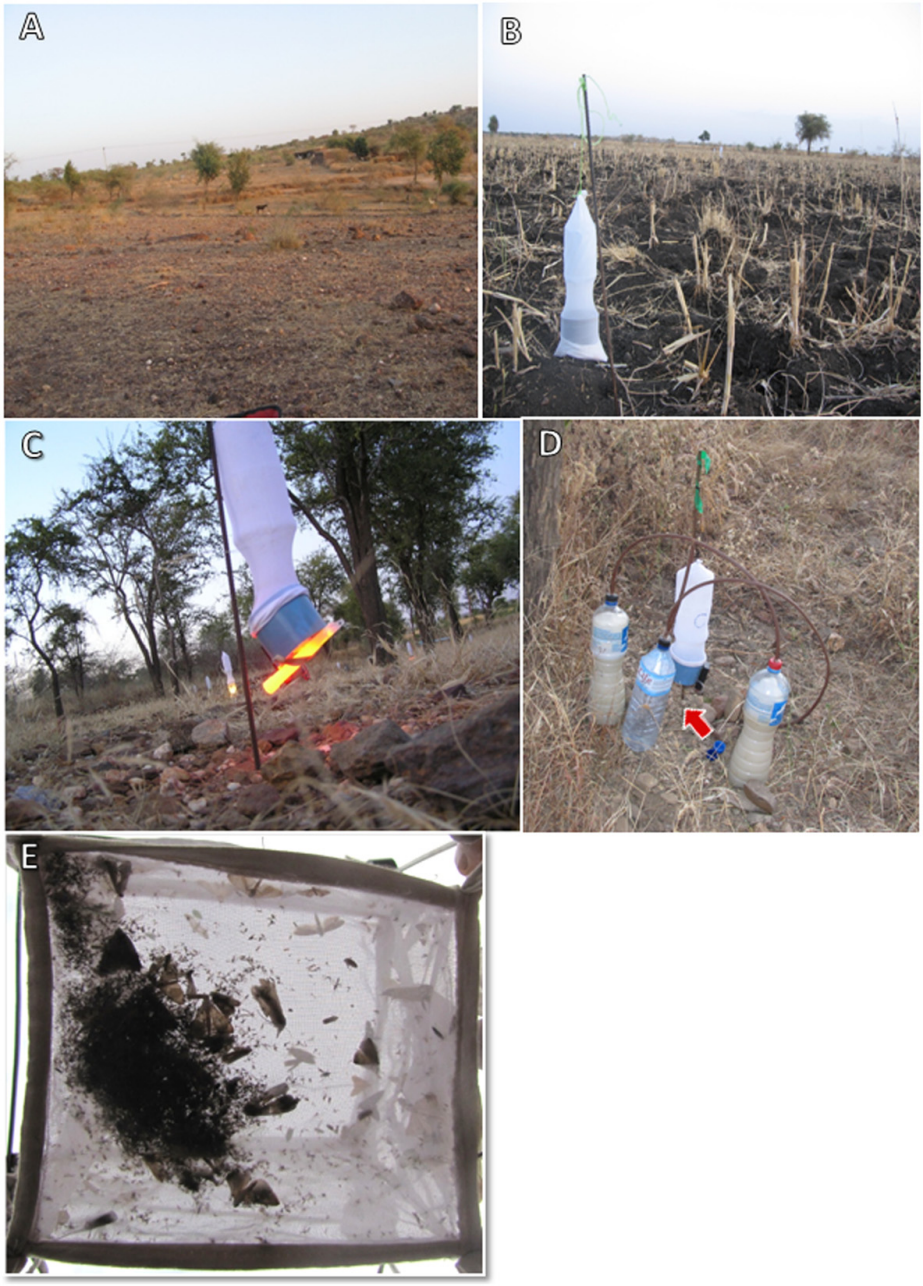
**(A-C) Field work in, Tigray, Northern Ethiopia: A) Rocky plain with sparse vegetation near the village of Ademiti. B)** Deeply cracked vertisol in a fallow sorghum field near the village of Gueza Meker showing a 3 V CDC trap. **C)** Sparse woodland near the village of Erdwayane with a 3 V trap baited with red light-stick. Note up-draft orientation of the traps. **D)** A 3 V CDC trap, baited with sugar-yeast mixture (SYM) producing CO_2_ as attractant. Note two bottles filled with SYM joined via an empty bottle serving to catch foam. Arrow points to the CO_2_ outlet under the trap which is deployed in the up-draft orientation. **E)** Typical catch of a down-draft 6 V incandescent light trap in Sheraro. To process and separate the sand flies from the rest of the insects in such catches can take up to an hour.

During the first year (November – December, 2011), experiments were carried out in three different habitats/locations: a) An arid plain with rocky terrain and sparse vegetation near the village of Ademeiti (Figure 1A) b) A fallow sorghum field with deeply cracked black cotton soil (=vertisol), near the village of Geza Meker (Figure 1B). c) A woodland dominated by *Balanites aegyptiaca* trees near the village of Erdweyane (Figure 1C).

During the second year, (February 2012) experiments were conducted in two different areas in Lemlem kebele with similar ecotopes, fallow Sorghum/sesame fields on vertisols: a) Gueza meker and, b) Gueza Adura.

### Traps and attractants

Experiments were conducted using CDC type suction traps deployed in the up-draft orientation with their opening about 15 cm above ground level. Suction traps deployed in the inverted (=updraft) orientation were previously shown to be more efficient at catching sand flies in open areas than traps deployed in the downdraft orientation [30]. In different experiments, traps were operated using either 6 V rechargeable batteries (standard CDC light traps) or two AA batteries (3 V) and augmented with one of five different means of attraction:

1. Six inch chemiluminescent light-sticks that give off light in the visible spectrum for 12 hours: red, yellow, green (ChemlightsTM, Caylume® West Springfield, MA 01089; Figure 1C).

2. Incandescent light trap (6 V, 150 mA, John W. Hock, Model 512, Gainesville, FL) powered by a 6 V lithium battery;

3. Fermenting sugar-yeast mixture (SYM). SYM was prepared using 12 g dehydrated Baker’s yeast purchased locally in Sheraro) and 150 g table sugar in 1 L of tap water. During the first year’s experiments traps were baited with two liters of SYM dispensed into two 1.5 L plastic soft-drink bottles that were interconnected with a plastic 6 mm gauge flexible tube via a third plastic bottle that was left empty to collect foam overflow (Figure 1D). The setup was similar to that previously described for trapping Anopheline mosquitoes [35].

In order to minimize trap/location/treatment bias, a Latin Square experimental design was adopted. Traps were placed 10 m apart and their position was rotated every night [38]. Due to logistical constraints, we were not able to test each attractant-trap combination in all possible locations [38]. During the first year’s experiments conducted in November – December of 2011, traps were arranged in a 5 × 5 matrix. Experiments were conducted for three nights and traps were deployed at 17:00 and collected at 06:00 the following morning. The total number of trap-nights (TNs) was 75 and divided as follows: 16TNs for the green light-stick, 18 TNs for the yellow light-stick, 16 TNs for the red light-stick, 14 TNs for the incandescent light and 11 TNs for the SYM. Due to logistical constraints, we conducted the experiment in 3 different locations one night per location.

Based on the first year’s results, during the second round of experiments (Feb 2012) only three attractants were compared. 1) Green light-stick. 2) Sugar/Yeast mixture (SYM). 3) 6 V 12 mAmp incandescent light. The Latin Square was reduced to a 3×3 matrix and experiments were conducted in two different locations simultaneously for three night. The total number of TNs was 18 for each attractant-trap combination. Due to a temporary shortage of yeast during the 2^nd^ year, only one liter of SYM in a single soft-drink bottle was used to bait each trap.

Distance between the traps in the Latin squares was approximately 15 m in order to minimize the possibility of traps competing with each other. Sand flies are attracted to light traps at distances that do not much exceed 2 m [28]. Distances of attraction to CO_2_ produced by SYM has not been determined experimentally but since the amounts of CO_2_ produced by the fermenting yeast are very small, we do not expect that effective attraction surpasses 6 m [29,36].

### Identification of sand flies

Sand flies collected were washed by placing them in a tea strainer inside a Petri-dish containing dilute (~1%) dish-washing detergent solution to remove hairs and other debris. Thereafter, flies were washed in tap water, transferred to a clean Petri-dish and counted under a stereo microscope. For identification, sand flies were mounted in Hoyer’s medium on microscope slides with their heads separate from thoraces. Identification of species was based on cibarial and pharyngeal armature as well as spermathecae of females and external genitalia of males [14,39-41].

### Data analysis

When data were distributed normally (normal distribution) a univariate analysis of variance (ANOVA) was used to compare the average yields of the different trap-attractant combinations. Least significant difference (LSD) post hoc analysis was utilized to ascertain the extent of the difference among the groups in cases where ANOVA was significant. The non-parametric equivalent test (Kruskal-Wallis) was used when trapping data did not conform to the normal distribution (e.g. aggregated). Sand fly numbers were log-transformed [log (n + 1)] to normalize the distribution and control the variance. This allowed the calculation of geometric means (Williams means, Mw) and the application of parametric tests [29,42-44]. For nonparametric comparisons, multiple-Mann-Whitney U tests were used and, P-values were adjusted with the Bonferroni correction to adjust for the inflation of type I errors when several Mann–Whitney tests are performed [38]. All statistical analyses were carried out using IBM SPSS statistics, version 19 for Windows and Microsoft® Office Excel 2007.

## Results

### First year experiments

**Figure 2:**
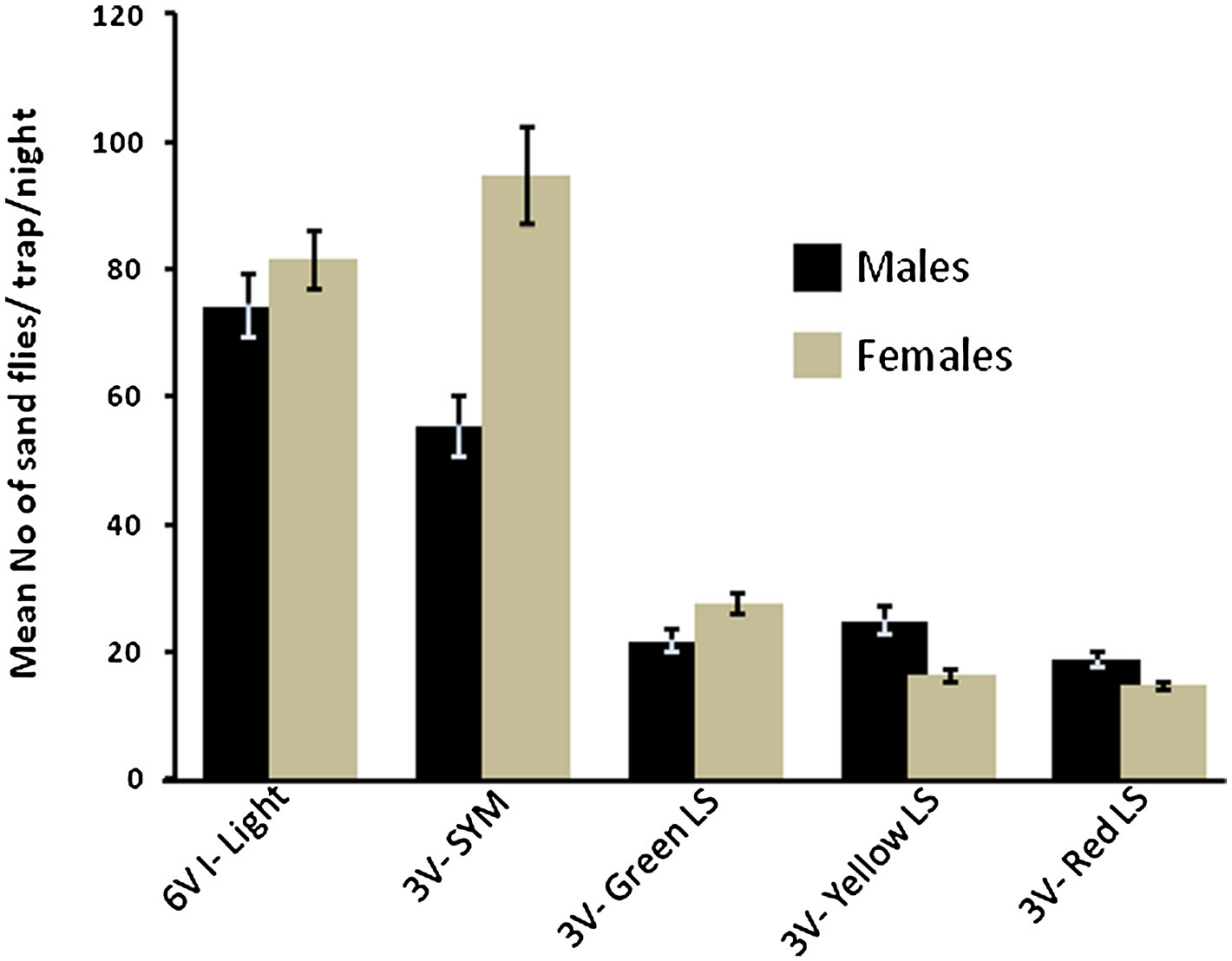
***Sergentomyia *spp. and *Phlebotomus *spp. caught by suction traps baited with different attractants.** Bars represent mean (±SE) number of sand flies (males black bars and females gray bars) per trap per night (11–16 trap nights per bar). There was no significant difference between the green, yellow and red chemical light-sticks (Kruskal-Wallis test, p > 0.05). Incandescent light (6 V, 150 mA) and Sugar-Yeast-Mixture (SYM), captured significantly more sand flies than the light-sticks.

(November – December, 2011)***:*** A total of 5,891 sand flies were caught over 3 nights in three different locations/environments. Of these, 2,741 were males and 3,150 were females (male/female ratio was slightly less than 0.9). There was a significant difference between the total numbers of sand flies captured during the three trap nights, each of which was conducted in a different location. During the 1^st^ night in the arid rocky plain with sparse vegetation 1,813 flies were captured. While 1,059 flies were captured in the fallow vertisol sorghum field during the 2^nd^ night. In the 3^rd^ night, significantly more sand flies (3,019) were trapped in the wooded area (Kruskal-Wallis test, p < 0.000). When averaged over the three nights, a significant difference was found between the different attractant/trap combinations (Kruskal-Wallis test, p < 0.05). Incandescent 6 V light traps were the most effective followed by SYM-baited traps (multiple-Mann-Whitney U tests, p < 0.05). Traps baited with different colored light-sticks captured significantly fewer flies (Figure 2, Table 1).

**Table 1:** Sand flies trapped in suction traps baited with different light sources or fermenting sugar solution

**Bait type (No of trap nights)**	**Mean ± SEM sand flies per trap/night**			**Total**
	**Total**	**♂♂**	**♀♀**	
6 V incandescent light (14)	155.71 ± 10.02	74.21 ± 5.02	81.5 ± 4.54	2,180
3 V SYM (11)	150.03 ± 12.82	55.35 ± 4.92	94.68 ± 7.56	1,651
3 V Green LS (16)	49.03 ± 3.58	21.55 ± 1.75	27.48 ± 1.68	785
3 V Yellow LS (18)	41.19 ± 3.33	24.88 ± 2.24	16.31 ± 0.98	742
3 V Red LS (16)	33.33 ± 2.11	18.72 ± 1.31	14.61 ± 0.64	533

Most traps operated on 3 V (2 AA batteries) except for the incandescent light trap that ran on 6 V (rechargeable lithium battery).

A sample of 1,365 (558 males and 807 females) sand flies was dissected and mounted on microscope slides for identification of species. The vast majority (~99%) belonged to the genus *Sergentomyia* and only a few were *Phlebotomus* spp. Eight different species of *Sergentomyia* were identified. *Sergentomyia (Parrotomyia) africana (*Newstead*),* Lewis, 1960 was by far the predominant species in most traps (45%), the only exception being SYM-baited traps where *S.(Sergentomyia) schwetzi* (Adler, Theodor & Parrot) comprised 75% of the catch, significantly divergent from the other attractant/trap combinations (ANOVA, F_*(df=8)*_ *=* 12.19, P < 0.000). Only few *Phlebotomus* spp. were collected without bias for any of the attractant/trap combination (Table 2).

**Table 2:** Different Sand fly species captured by updraft suction traps baited with incandescent light, Green, Yellow and Red light-sticks and sugar-yeast mixture (SYM)

	**6 V incandescent light (14 TNs)**		**3 V sugar yeast mixture (11 TNs)**		**3 V green light-stick (16 TNs)**		**3 V yellow light-stick (18 TNs)**		**3 V red light-stick (16 TNs)**	
	**♂♂**	**♀♀**	**♂♂**	**♀♀**	**♂♂**	**♀♀**	**♂♂**	**♀♀**	**♂♂**	**♀♀**
*Phlebotomus spp.*	12	3	0	8	4	3	0	6	0	5
**Total (%) *Phlebotomus***	**15 (0.6)**		**8 (0.4)**		**7 (0.8)**		**6 (0.8)**		**5 (0.9)**	
*S. africana*	665	610	44	68	258	256	372	169	193	99
*S. bedfordi*	3	28	0	6	0	3	0	0	0	11
*S. adleri*	3	15	3	19	0	0	0	7	3	0
*S. squamipleuris*	90	137	6	0	7	21	11	14	24	10
*S. schwtezi*	52	72	496	777	4	17	17	4	19	20
*S. clydei*	0	52	0	66	3	21	0	26	0	9
*S. antennata*	116	42	3	24	50	54	26	22	57	34
*S. minuta (complex)*	98	182	58	73	20	64	21	47	3	46
**Total (%) *Sergentomyia***	**2,165 (99.2)**		**1,643 (99.5)**		**778 (99.1)**		**736 (99.05)**		**528 (99.06)**	
**Total**	**2,180**		**1,651**		**785**		**742**		**533**	

TN = Trap Night.

### Second year experiments

(February 2012): A total of 3,879 (Males: 2,270; Females: 1,609) sand flies were caught during three trapping nights (totaling 54 TNs) in two locations simultaneously. The male/female ratio was 1.4 and no significant difference was found between the different attractants (Kruskal-Wallis test, p < 0.05). For identification, 2,561 flies (1,400 males and 1,161 females) were mounted in Hoyer’s medium.

**Figure 3:**
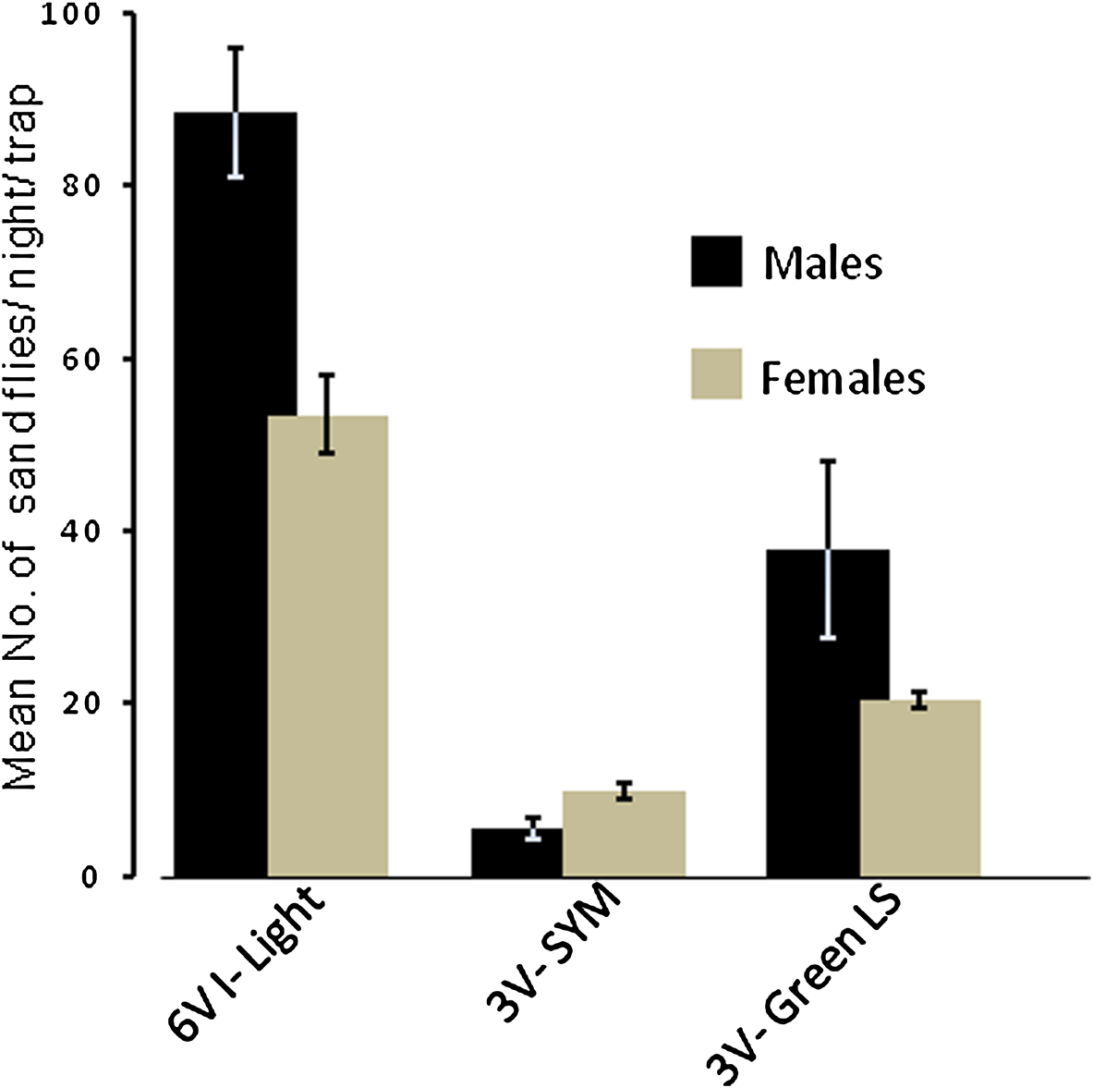
**Male and female sand flies caught by suction traps with different baits.** Bars represent mean (±SE) number of sand flies (males separately from females) per trap per night (totaling 18 trap nights for each attractant-trap combination).

There was no significant difference between the mean numbers of sand flies captured in the two locations sampled (Mann-Whitney *U* test, p > 0.05). However, a significant difference was found between the different attractant-trap combinations (Kruskal-Wallis test, p < 0.05). The 6 V incandescent light traps were the most effective (mean of 141.94 sand flies per TN), followed by green light-sticks -baited 3 V traps (mean of 58.11 sand flies per TN). Finally, SYM-Baited 3 V traps yields were almost four- fold lower than in the latter (mean of 15.44 sand flies per TN, Multiple - Mann Whitney *U* test, p < 0.01, Table 3, Figure 3).

**Table 3:** Sand flies captured by suction traps baited with different attractants during the second year trials (18 TNs each - February 2012)

**Bait type**	**Mean (SEM) sand flies/trap/night**			**Total for 18 trap nights**
	**Total**	**♂♂**	**♀♀**	
**6 V incandescent light**	141.94 ± 11.94	88.50 ± 7.50	53.44 ± 4.44	2,555
**3 V SYM**	15.45 ± 0.44	5.56 ± 1.33	9.89 ± 0.89	278
**Green LS**	58.08 ± 9.33	37.72 ± 10.28	20.39 ± 0.94	1,045

There was a significant difference in the sand fly species composition between traps with different baits (ANOVA, F _*(df =10)*_ = 6.7, P < 0.05). Overall, *Sergentomyia* spp. vastly outnumbered *Phlebotomus* spp. by about 9:1 in the incandescent light traps and the green light-stick traps where *S. africana* was the most common species comprising 74.39% of the catch. However, in the SYM-baited traps *Phlebotomus* spp. were more numerous and *P. orientalis* comprised 40.28% of the entire catch. *S. schwetzi* (26.61%), *P.*(*Parvidens*) *spp* (9.71%) and *S. africana* (17.26%) were also common in the SYM baited traps (Table 4).

**Table 4:** Sand fly species captured by updraft suction traps baited with different attractants

**Species**	**6 V incandescent light**		**3 V green light-stick**		**3 V sugar yeast mixture**	
	**♂♂**	**♀♀**	**♂♂**	**♀♀**	**♂♂**	**♀♀**
*P. papatasi*	0	0	1	2	0	1
*P. orientalis*	89	71	55	26	37	75
*P. rodahini*	0	0	0	1	2	1
*P.* (*Parvidens*)spp*.	48	70	8	13	15	12
**Total (%) *Phlebotomus***	278 (10.8)		106 (10.1)		143 (51.4)	
*S. africana*	1,252	745	552	288	28	19
*S. bedfordi*	0	9	0	0	0	0
*S. adleri*	0	0	1	1	0	0
*S. squamipleuris*	5	20	1	4	0	0
*S. schwetzi*	53	65	26	9	15	59
*S. clydei*	11	21	8	10	1	6
*S. antennata*	37	59	24	16	1	6
**Total (%) *Sergentomyia***	2,277 (89.1)		940 (89.9)		135 (48.9)	
**Total**	2,555		1,046		278	

*P.(*Parvidens*) *heischi & P.*(*Parvidens*) *lesleyae* were not separated to species level.

Total numbers captured during 18 trap nights. A higher percentage of *Phlebotomus* spp. and more females thereof, were attracted to the SYM-baited traps with CO_2_ emanating from the fermenting sugar.

## Discussion

The current study evaluated readily available, low cost means for monitoring phlebotomine sand flies in remote region of northern Ethiopia. The initial phase of the experiments established that chemiluminescent light-sticks were attractive to sand flies confirming previous studies [45]. Moreover, there was no significant difference between the different colors (=emitted wave lengths) light-sticks that were tested (Table 1, Figure 2).

These experiments also verified that emanations from fermenting sugar yeast mixtures (SYM) can be used as a source of CO_2_ to attract and trap chiefly mammal-biting sand flies (most *Phlebotomus* spp. and certain *Sergentomyia* spp. [e.g. *S. schwetzi*]) (Table 1, Figure 2). Since there were no significant differences between the different colors, during the second phase of the study we repeated the experiments using only green light-sticks, incandescent light and SYM. Unfortunately, due to a temporary shortage of bakers’ yeast in the local market in Sheraro which happened to coincide with our study; we had to reduce the amount of SYM to 1 liter per trap per night. As a result, the yield of SYM-baited traps declined dramatically while the incandescent light traps and the green light-stick-baited traps caught similar numbers of sand flies as they had done during the initial round of experiments (compare Figures 2 and 3). However, SYM-baited traps captured over 50% *Phlebotomus* spp. while the light traps captured only circa 10% (Table 4). Moreover, SYM-baited traps favored the females of mammal-biting *Phlebotomus* spp., the more important faction of the population required for determining infection rates with *Leishmania*. Specifically, *P. orientalis* females, the probable vectors of VL in the region, were as numerous in 3 V SYM-baited traps as in the 6 V light traps despite the fact that the latter traps captured ten times as many sand flies over all (Table 4).

6 V light traps that are designed for deployment in the down draft orientation typically capture an abundance of phototropic insects including tiny dipterans and many medium sized hymenopterans, lepidopterans, coleopterans etc. (Figure 1E). Such yields can be extremely laborious to process and in many cases the sand flies are damaged by the larger heavier insects. In our study, deployment of such traps in the up-draft position and use of lower voltage (3 V) reduced the number of heavier insects because the weaker pull of the motor combined with the upward direction, allowed the larger, stronger insects to escape [30].

A further improvement was achieved by replacing the light source with SYM. The components of SYM, sugar and yeast were generally (unfortunately not always) available in the market in Sheraro. In February, at the height of the *Phlebotomus* season, SYM-baited traps yielded a high percentage (51%) of *Phlebotomus* spp. and captured more *P. orientalis*, the vector of VL in Sheraro, than other attractant-trap combinations. The amount of CO_2_ released by SYM was apparently very important as demonstrated by the much lower catch obtained during the second year when only one liter of SYM was used instead of the 2 L used during the first year (compare Figures 2 and 3). Therefore, we recommend using larger volumes (2–3 liters per trap) of SYM keeping the approximate ratio of dry Bakers’ yeast to sugar to water at approximately 12:150:1000 w/w/v.

## Conclusions

Monitoring phlebotomine sand flies using inverted up-draft traps has the advantage of catching mostly small fragile insects such as sand flies and fewer heavy ones that damage the catch. Suction traps utilizing 2 AA batteries and a light-stick weigh approximately 350 g including the collection cage. This is a big advantage over 6 V incandescent light traps that require heavy batteries weighing 1.5 kg each, especially in places where deployment of traps may require lengthy walks over uneven terrain. Three volt (3 V) traps constitute appropriate means for monitoring sand flies in remote areas despite the fact that the yield of sand flies is probably lower than that of 6 V traps (Faiman, unpublished observations). Therefore, it may be necessary to deploy more 3 V traps to catch the same number of sand flies as with 6 V traps. Using SYM-baited traps does necessitate carrying relatively heavy liquid-filled bottles. However, the SYM baited traps specifically capture the blood-seeking females of hematophagous insects, thereby saving much of the work associated with processing the entomological yields of these traps. Our findings identified two different means for monitoring sand flies in remote inaccessible areas characteristic of many leishmaniasis foci. We also report for the first time on the phlebotomine sand fly fauna of the district of Sheraro a remote VL focus in northern Ethiopia.

## Competing interests

The authors declare that they have no competing interests.

## Authors’ contributions

ODK: Conceived the study, contributed to experimental design, performed the experiments, processed the trap yields, identified the sand flies, analyzed the data, wrote the manuscript. RF: Contributed to experimental design, performed the experiments, analyzed the data. AGS: Performed the experiments, processed the trap yields, identified the sand flies, AH: Conceived the study, provided logistical and scientific support for the study. TGM: Contributed to experimental design, performed the experiments, consulted on identification of the sand flies, assisted in writing the manuscript. AW: Conceived the study, contributed to experimental design, performed the experiments, wrote the manuscript. All authors read and approved the final version of the manuscript.
